# Long-Term Outcome of Individuals with Borderline Thrombocytopenia

**DOI:** 10.1371/journal.pmed.0030093

**Published:** 2006-01-17

Many cases of thrombocytopenia in adults are diagnosed incidentally after a routine blood cell count; more efficient laboratory processes have meant that an increasing number of asymptomatic individuals with borderline low platelet counts (100 × 10 ^9^/l to 150 × 10 ^9^/l) are now being identified. There are gaps in our knowledge of this condition, and the natural history of thrombocytopenia has not been systematically studied. For example, it's not known how many people might develop other diseases associated with a low platelet count, or how many people can live outwardly healthy lives with borderline normal platelet counts. In the absence of other diseases, current treatment guidelines are based on expert opinion rather than on randomized controlled trials, and patients are treated if they have severe bleeding and/or extremely low platelet counts.

One cause of an asymptomatic low platelet count is an immune thrombocytopenia (ITP), either primary (idiopathic) or secondary to an autoimmune disorder. Hence, thrombocytopenia might be the start of a systemic autoimmune disease, which can make it difficult to diagnose secondary immune thrombocytopenia from ITP. Systemic autoimmune diseases could be treated if detected early.

In this month's *PLoS Medicine*, Roberto Stasi and colleagues followed the natural history of 260 apparently healthy adults who had low platelet counts and were considered to have “borderline thrombocytopenia.” After six months of monitoring to see if the condition persisted, 191 patients were monitored for, on average, 64 months. In 64% of these patients, thrombocytopenia resolved spontaneously or persisted with no other disorders becoming apparent. In the remainder, the most frequent event was the development of an autoimmune disease. The 10-year probability of developing idiopathic ITP was 6.9% and of developing autoimmune disorders other than ITP was 12%. Among 36 patients (14%) with stable normal platelet counts, most (23/36) improved their counts during the initial six-month assessment. This pattern might be because they had a “silent” viral infection or a minor insult of a different nature to the bone marrow, the authors suggest. Another theory is that these patterns might also be due to seasonal variation of the platelet count.

Interestingly, in this study many patients with a low platelet count who developed autoimmune disease did not have conventional markers of autoimmunity. This finding conflicts with previous work that has suggested that autoantibodies are present many years before the diagnosis of systemic lupus erythematosus. Specifically, two patients in this study who developed systemic lupus erythematosus did not have detectable autoantibodies.

Many patients with low platelet count also had chronic thyroiditis. Based on previous evidence, the relationship between autoimmune thrombocytopenia and chronic thyroiditis is controversial, and the occurrence of patients with chronic thyroiditis with borderline thrombocytopenia (7%) was not higher than the general population (6.2%). Patients with chronic thyroiditis did not have a higher risk of developing ITP or other autoimmune disorders than the other individuals, the authors say.

This study will improve clinicians' understanding of the natural history of borderline thrombocytopenia, although exactly what these figures mean for individuals is hard to judge because there was no control group. The authors acknowledge that much more needs to be done. For example, for conditions such as ITP, diagnosis is still by exclusion, so other diseases that might cause thrombocytopenia have to be ruled out. Further work will need to be done to establish whether the risk for these patients of developing an autoimmune disease is higher than in the general population and, most practically, whether intensive follow-up has a positive impact on patients' prognosis.

